# Supplementary material for: A flexible topo-optical sensing technology with ultra-high contrast
Source: Nat Commun. 2020 Mar 19;11:1448. doi: 10.1038/s41467-020-15288-8 (PMC7081276; doi:10.1038/s41467-020-15288-8)
Supplement: Supplementary file 1 — Supplementary Information [file 41467_2020_15288_MOESM1_ESM.pdf]

Supplementary Information

A Flexible Topo-optical Sensing Technology with Ultra-high Contrast

Wang et al.

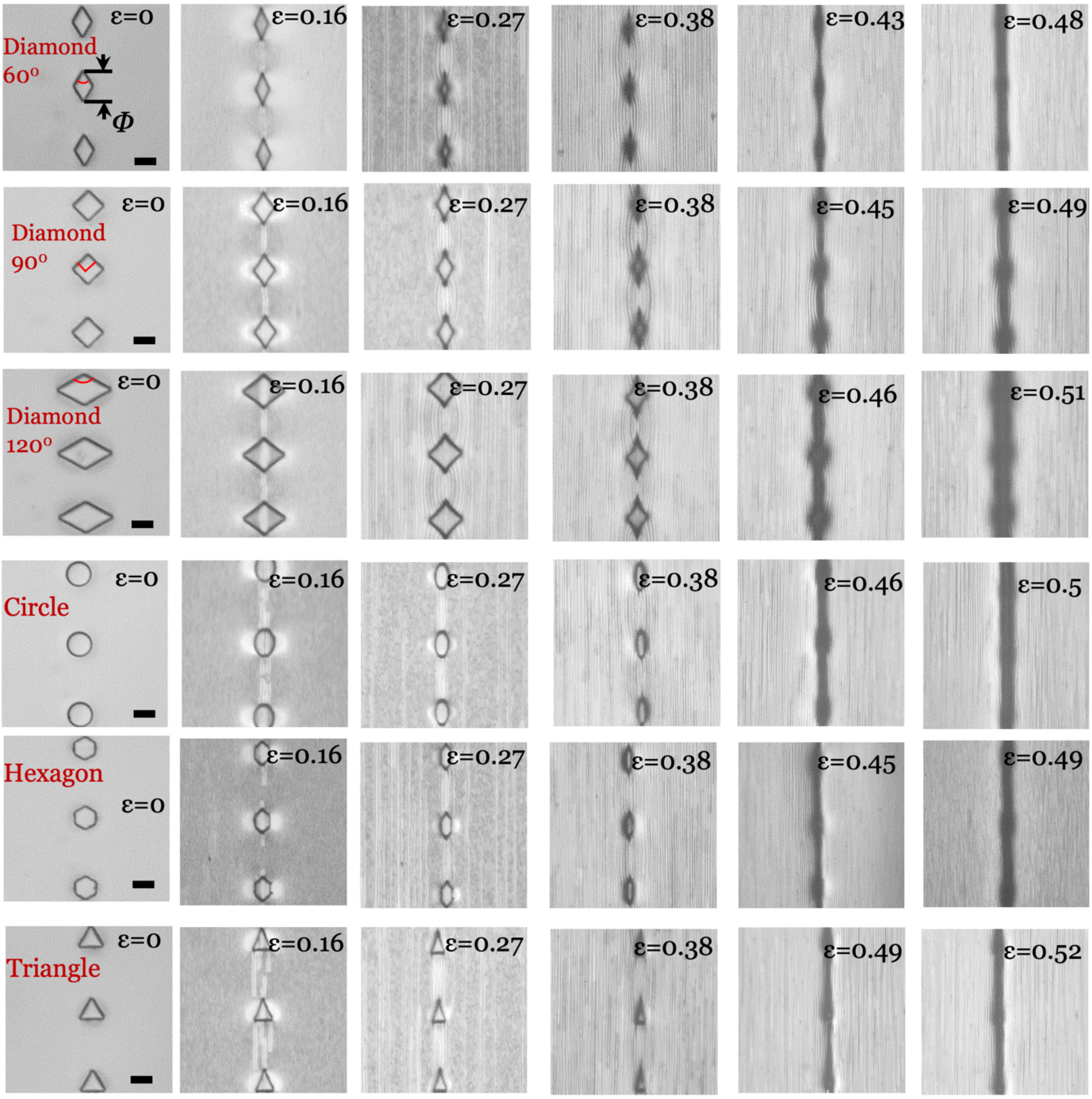

Supplementary Figure 1. The formation of folding guided by the single line lattice pattern with different pattern shape, scale bar = 40 μm.

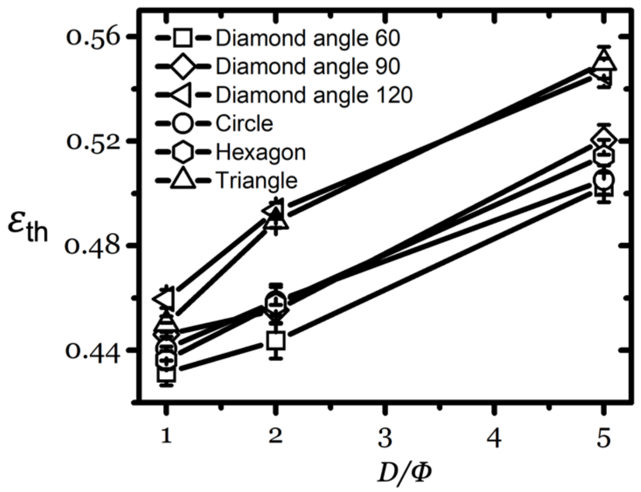

Supplementary Figure 2. Summary of threshold strains to achieve targeted folding on the surface patterned with different shape.

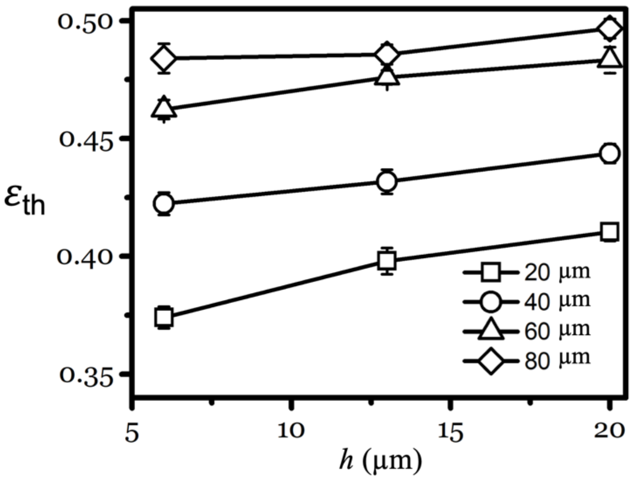

Supplementary Figure 3. Summary of threshold strains to achieve targeted folding with dependence on the hole depth.

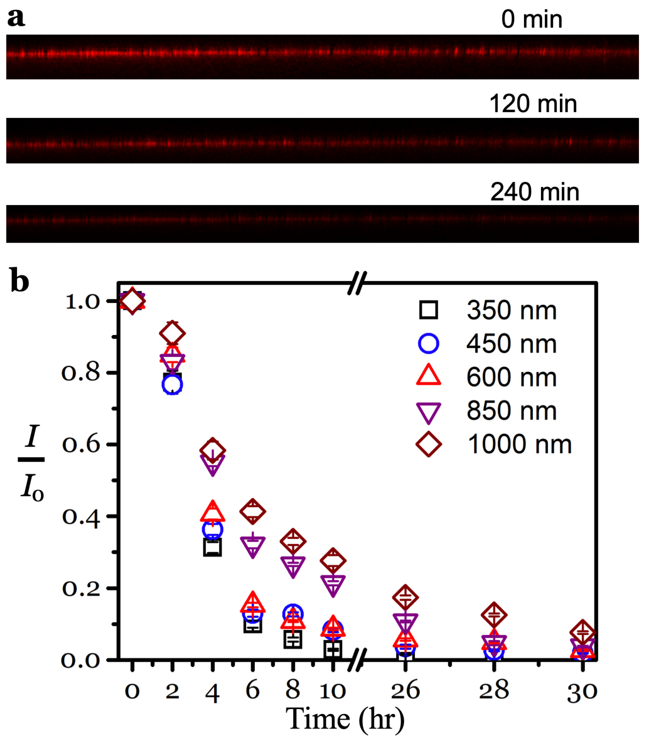

Supplementary Figure 4 (a). The quenching observation for an Ir-III film of 450 nm; (b) Quenching kinetics analysis Ir-III films coated on PDMS surface with different thickness in the open air.

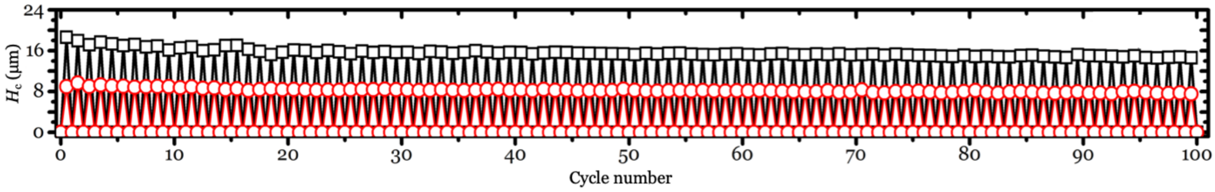

Supplementary Figure 5 The cyclic testing results (up to 100 cycles) for samples with Φ = 80 μm (□) and Φ = 40 μm (○), at D/Φ =2.

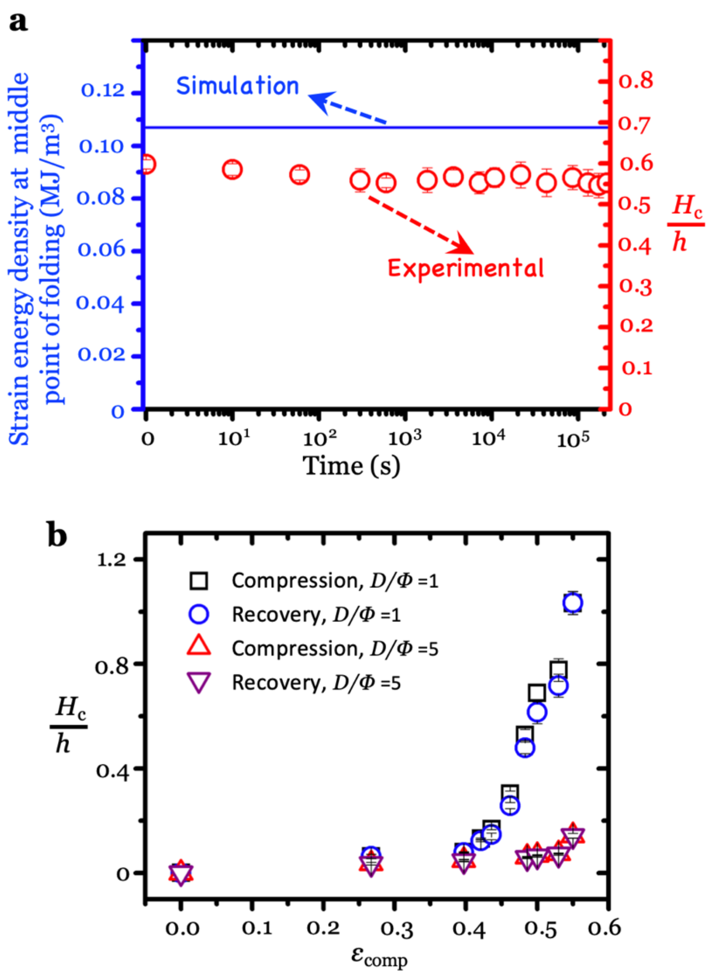

Supplementary Figure 6 (a). Comparison the simulation and experimental results for relaxation behaviour under a progressive $\varepsilon_{\text{comp}}$ =0.5 for the surface with a single micro-hole array (Φ = 40 μm, D/Φ=5). (b) The hysteresis results for targeted folding depth on the surface with a single micro-hole array (Φ = 40 μm, D/Φ=1 and D/Φ=5).

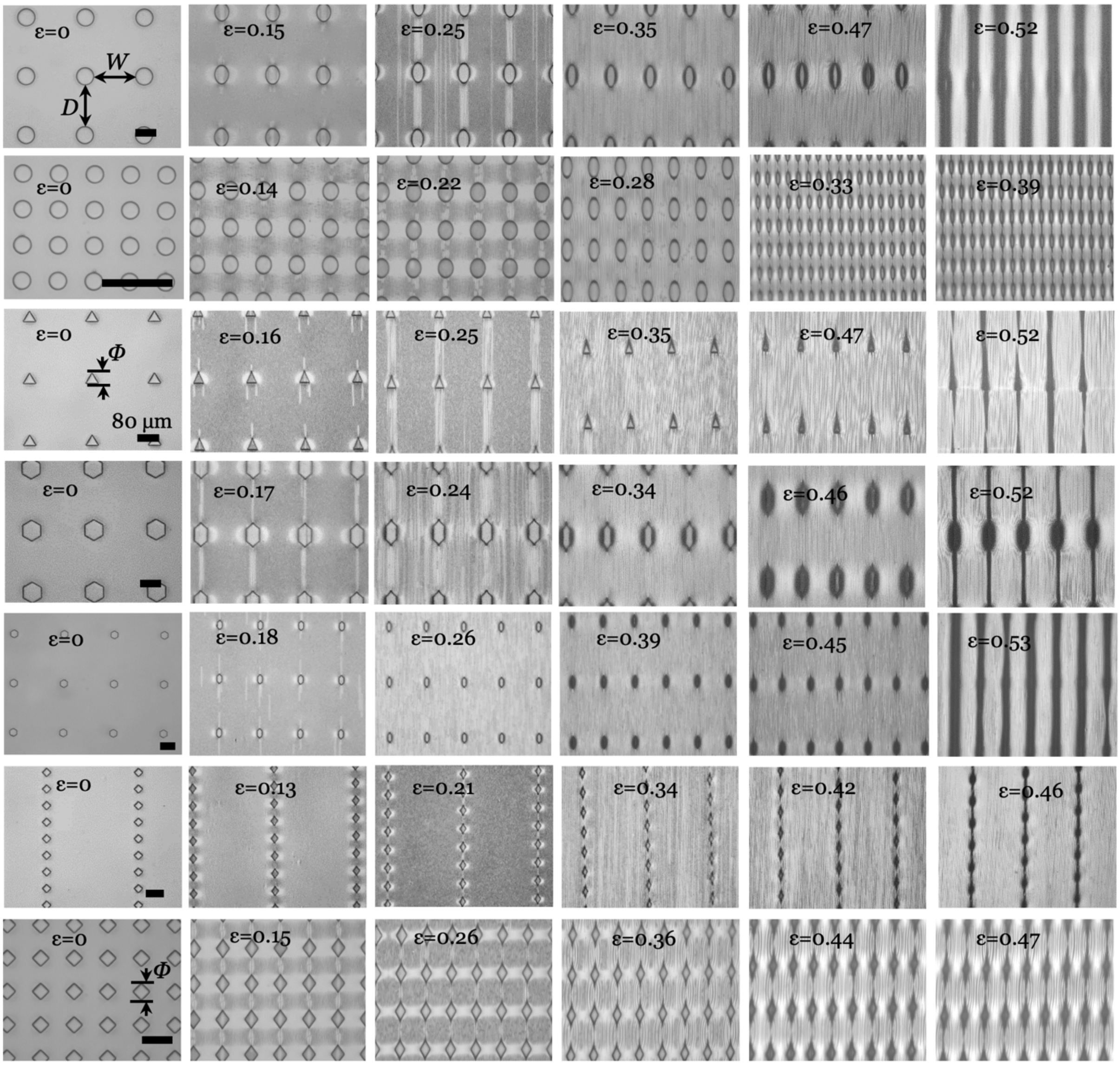

Supplementary Figure 7 The formation of folding guided by the square lattice pattern with different pattern shape, W/D, and D/Φ.

.

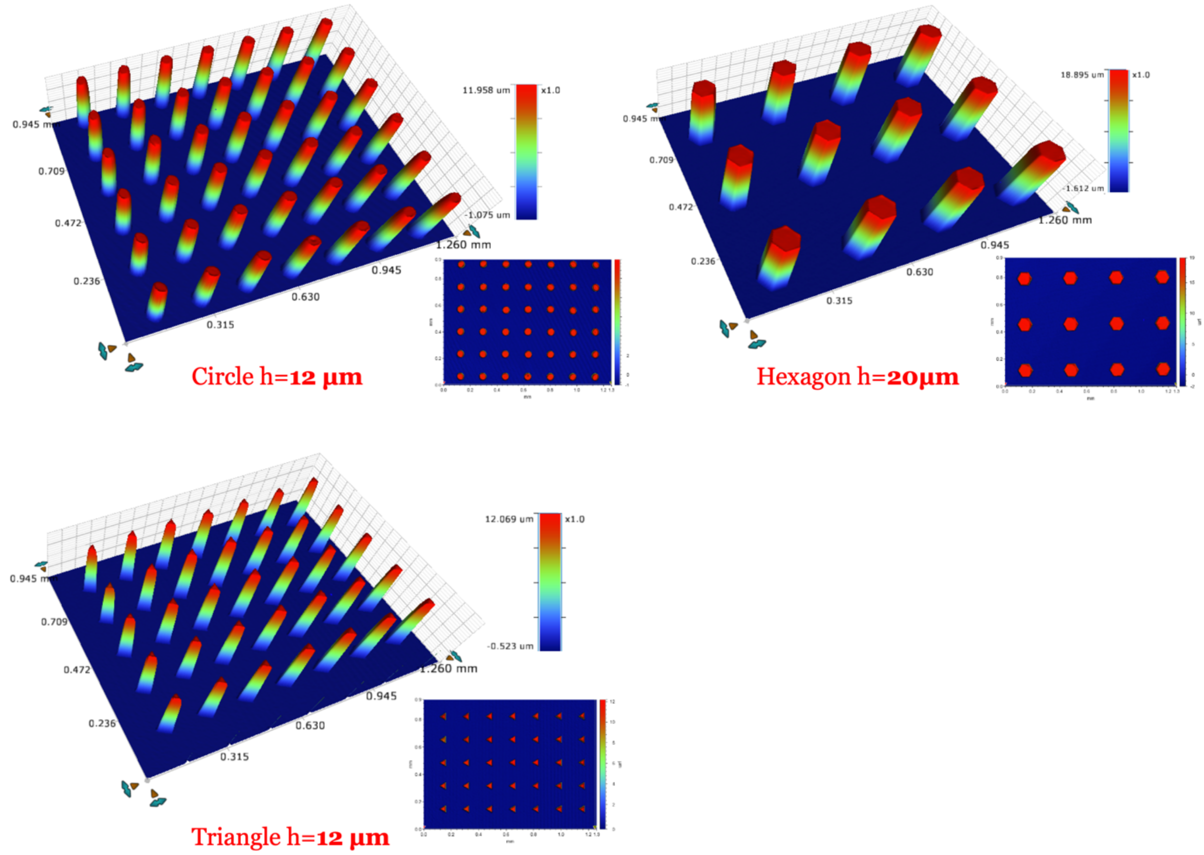

Supplementary Figure 8 Profiling of the fabricated SU-8 array templates with different geometrical factors.

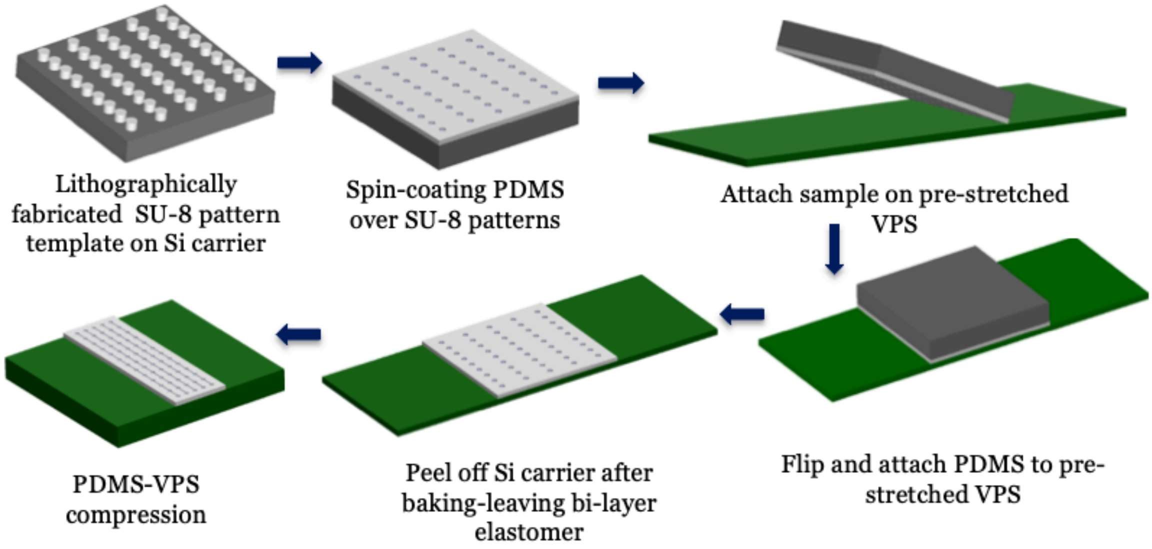

Supplementary Figure 9 The schematic illustration of fabrication process of structural surface by spin-coating a thin PDMS precursor layer on a lithographically made template.
