# Supplementary material for: A flexible topo-optical sensing technology with ultra-high contrast
Source: Nat Commun. 2020 Mar 19;11:1448. doi: 10.1038/s41467-020-15288-8 (PMC7081276; doi:10.1038/s41467-020-15288-8)
Supplement: Supplementary file 3 — Description of Additional Supplementary Information Files [file 41467_2020_15288_MOESM3_ESM.pdf]

Description of Additional Supplementary Files

File Name: Supplementary Movie 1
Description: Targeted folding on the surface patterned with single-line of micro-hole array (Φ = 60 µm, D = 120 µm, h = 12 µm).

File Name: Supplementary Movie 2
Description: Finite element simulation of In-plane strain energy localization for the surfaces with a single micro-hole array (Φ = 40 µm) of D/Φ =1 and D/Φ =5.

File Name: Supplementary Movie 3
Description: Finite element simulation of out-of-plane strain energy localization and mophological development for the surfaces with a single micro-hole array (Φ = 40 µm) of D/Φ =1 and D/Φ =5.

File Name: Supplementary Movie 4
Description: Demonstration of targeted folding on complicated surface patterned with lattice array (multi-line of micro-hole array).

File Name: Supplementary Movie 5
Description: Demonstration of an in-plane strain sensor with programmable stepwise logic sensing function.
